# Gastric Alimetry^®^ Test Interpretation in Gastroduodenal Disorders: Review and Recommendations

**DOI:** 10.3390/jcm12206436

**Published:** 2023-10-10

**Authors:** Daphne Foong, Stefan Calder, Chris Varghese, Gabriel Schamberg, William Xu, Charlotte Daker, Vincent Ho, Christopher N. Andrews, Armen A. Gharibans, Greg O’Grady

**Affiliations:** 1 School of Medicine, Western Sydney University, Campbelltown, NSW 2560, Australia; 2 Department of Surgery, Auckland City Hospital, Auckland 1023, New Zealand; 3 Alimetry Ltd., Auckland 1010, New Zealand; 4 Auckland Bioengineering Institute, University of Auckland, Auckland 1010, New Zealand; 5 Department of Gastroenterology, North Shore Hospital, Auckland 0620, New Zealand; 6 Department of Gastroenterology and Hepatology, Campbelltown Hospital, Sydney, NSW 2560, Australia; 7 Division of Gastroenterology, Cumming School of Medicine, University of Calgary, Calgary, AB T2N 1N4, Canada

**Keywords:** gastroparesis, chronic nausea and vomiting, functional dyspepsia, body surface gastric mapping, disorders of gut brain interaction, motility disorders

## Abstract

Chronic gastroduodenal symptoms are prevalent worldwide, and there is a need for new diagnostic and treatment approaches. Several overlapping processes may contribute to these symptoms, including gastric dysmotility, hypersensitivity, gut–brain axis disorders, gastric outflow resistance, and duodenal inflammation. Gastric Alimetry^®^ (Alimetry, New Zealand) is a non-invasive test for evaluating gastric function that combines body surface gastric mapping (high-resolution electrophysiology) with validated symptom profiling. Together, these complementary data streams enable important new clinical insights into gastric disorders and their symptom correlations, with emerging therapeutic implications. A comprehensive database has been established, currently comprising > 2000 Gastric Alimetry tests, including both controls and patients with various gastroduodenal disorders. From studies employing this database, this paper presents a systematic methodology for Gastric Alimetry test interpretation, together with an extensive supporting literature review. Reporting is grouped into four sections: Test Quality, Spectral Analysis, Symptoms, and Conclusions. This review compiles, assesses, and evaluates each of these aspects of test assessment, with discussion of relevant evidence, example cases, limitations, and areas for future work. The resultant interpretation methodology is recommended for use in clinical practice and research to assist clinicians in their use of Gastric Alimetry as a diagnostic aid and is expected to continue to evolve with further development.

## 1. Introduction

Chronic gastroduodenal symptoms are common globally, and present significant quality of life and socioeconomic burdens. Over 10% of people worldwide are affected by chronic gastroduodenal symptoms, with >7% affected by functional dyspepsia alone [1]. Chronic nausea and vomiting syndrome (CNVS) and gastroparesis (Gp) are additional debilitating conditions, together recognised as nausea and vomiting syndromes (NVS), with a combined global prevalence of ~1% [2,3,4]. However, differentiating and diagnosing these disorders is challenging due to their overlapping symptom and testing profiles [5,6]. There is a pressing need to advance diagnostic testing in these conditions in order to advance personalized targeted therapy.

Gastroduodenal symptoms may arise from a variety of abnormalities including disordered motility, visceral hypersensitivity, immune activation, gastric outflow resistance, and brain–gut axis dysregulation [7,8,9]. However, there is a lack of diagnostic biomarkers to differentiate which of these abnormalities in isolation or combination may contribute to an individual patient’s presentation. Measuring gastric emptying with either scintigraphy or a breath test is currently the only widely available test of gastric function. Gastric emptying testing (GET) has been used to define gastroparesis historically and may inform therapy when delayed. However, limitations to GET include potentially labile results over time and insensitivity to neuromuscular pathologies [5,6,10,11]. Antroduodenal manometry is an additional prominent diagnostic test of gastroduodenal function but is now infrequently used due to its invasiveness, while other tests mainly occupy research niches [12]. 

Gastrointestinal peristalsis is coordinated by an underlying gastric electrical activity generated by the interstitial cells of Cajal (ICC) [13]. Additional influences from smooth muscle, extrinsic and enteric nervous systems, and neurohormonal feedback provide critical co-regulatory inputs that enable effective meal responses, trituration and emptying [14]. Historically, electrogastrography (EGG), was introduced as a clinical tool for assessing gastric myoelectric activity using a small number of cutaneous electrodes [15]. Although it is non-invasive and easy to administer, EGG had multiple limitations that hindered clinical utility and adoption, including poor specificity for normal vs. disease states in individual patients, and a lack of clear impact on clinical care [15,16].

**Figure 1:**
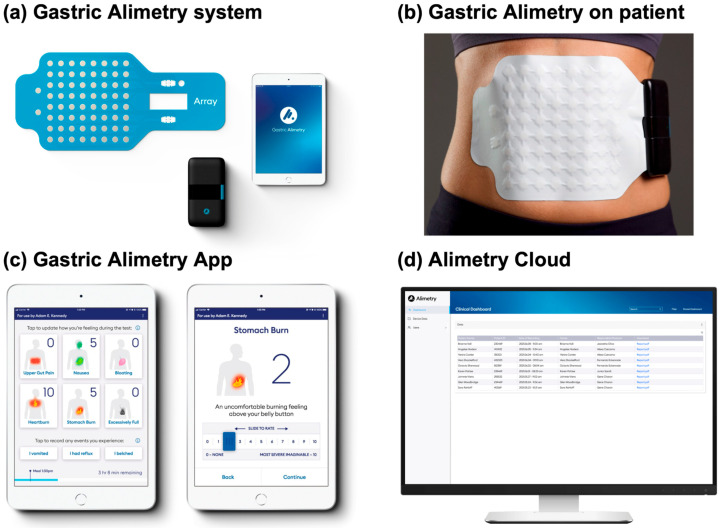
Gastric Alimetry setup. (**a**) Gastric Alimetry device consisting of a high-resolution electrode array (8 × 8 pregelled Ag/AgCl electrodes), Alimetry Reader and Gastric Alimetry app; (**b**) Device positioned over the epigastrium; (**c**) Gastric Alimetry App consisting of symptom logging; (**d**) Alimetry Cloud where clinicians can access and store Gastric Alimetry patient reports.

Gastric Alimetry^®^ (Alimetry Ltd., Auckland, New Zealand) is a new test of gastric function for non-invasively assessing gastric motility using simultaneous body surface gastric mapping (BSGM) [17] and validated symptom profiling [18] (Figure 1). BSGM is a high-resolution method of assessing gastric electrical activity, employing dense electrode arrays to measure and map human gastric slow-wave activity non-invasively (Figure 1a,b) [17,19,20,21]. A standard 4.5 h test consists of a fasting pre-prandial recording (30 min), meal (10 min) and post-prandial recording (4 h) [22]. Signals are filtered, processed and visualised using a validated automated pipeline [23], then analysed with novel metrics that offer numerous significant advances over previous EGG approaches [22,24]. The system also includes a validated app for tracking patient-reported symptoms (Figure 1c) [18], providing a second essential layer of data that are complementary to the electrophysiological analysis [25]. Altogether, this system provides a new tool to assess and interpret gastric function and symptom profiles in various gastroduodenal disorders.

Our research collaborations have accumulated a database of over 2000 Gastric Alimetry tests encompassing healthy volunteers as well as a wide variety of clinical disorders which has been used to publish several case series in diverse disorders including CNVS, gastroparesis, type 1 diabetes, functional dyspepsia, and post-gastric surgery [17,20,22,26,27,28,29]. We have established the first phenotype set that is based on proposed mechanisms of gastric activity and symptom generation [30]. This has been further supported by comparisons to other conventional diagnostic tests such as EGG [16] and GET [31], as well as in its potential to guide patient-specific therapy [32].

Based on this database and a growing BSGM literature, we present here a systematic technical approach to test interpretation for Gastric Alimetry, together with a supporting literature review that provides the evidence for its use in clinical practice. The purpose of the article is to present this clinical report interpretation framework, with references and a review of supporting literature, to propose a standardized approach for clinicians to adopt in their diagnostic work-up of patients. Case examples are presented, along with discussion of limitations and ongoing areas of research. It is also anticipated that this test interpretation system will form a valuable resource for the scientific community and will be iteratively improved as further evidence becomes available, and as the test evolves.

## 2. Overview of Test Interpretation Methodology

The analysis of the report typically encompasses four primary sections:

- Test Quality
- Spectral Analysis
- Symptoms
- Conclusions

Each section is discussed below, with reference to relevant literature, before concluding with the suggested reporting format, and example cases. Since a comprehensive literature search of gastric mapping and EGG has previously been conducted [25], the purpose of our current work was to utilize this literature to support the proposed test interpretation methodology. A first classification scheme of eight objective phenotypes arising from Gastric Alimetry has recently been introduced by the BSGM Working Group, which offers further guidance for the Gastric Alimetry test interpretation [25,30]. As more clinical evidence is collected, it is expected that this classification scheme will be further updated and refined.

## 3. Step 1: Assess Test Quality

**Figure 2:**
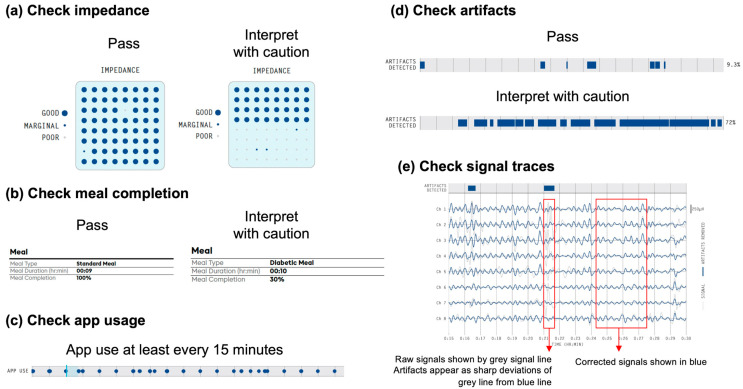
Summary of ‘Test Quality’ guidelines. (**a**) Checking impedance for electrode signal quality is ‘good’ for at least half the electrodes; (**b**) Checking meal completion is above 50%; (**c**) Checking proportion of artifacts is less than 50%; (**d**) Checking app usage was at least every 15 min; (**e**) Checking raw signal traces for uncertainties in artifacts.

The first step in test interpretation is to check test technical quality, as outlined in Figure 2. Gastric signals are low in amplitude, being two orders of magnitude weaker than cardiac signals, such that rigorous attention to test methods and quality is essential [25,33]. Guidance on test quality interpretation is provided by the Gastric Alimetry Report Guidelines [34], which are adopted here.

- Checking impedance (Figure 2a)

The impedance of the skin–electrode interface is a key determinant of signal quality [17,35]. As the Gastric Alimetry App only allows a test to be commenced if impedance is sufficient, with alerts then actioned if impedance rises above the threshold, test quality failures due to poor impedance are rare. If signal quality (good/marginal/poor) is ‘good’ for at least half of the electrodes, this is considered a pass, with marginal electrodes considered acceptable. However, if signal quality is marginal or poor across a majority of channels, then the test should be interpreted with caution. The key risk in this context is that motion artifacts may be accentuated in the presence of poor impedance.

- Checking meal completion (Figure 2b)

The standard meal for a Gastric Alimetry test is currently a 482 kcal meal consisting of an oatmeal bar and a nutrient drink. If meal completion is <50%, the test should be interpreted with caution. This determination was based on a sensitivity analysis revealing that a half-sized portion was sufficient to trigger meal responses and reliably detect dysrhythmic phenotypes using Gastric Alimetry [27]. While further research is needed to evaluate the effect of meal compositions and sizes on test metrics, low meal completion may compromise metric interpretations, in both spectral analysis and symptom generation [36]. In practice, we find the high majority of patients can complete >50% of the standard meal, even in the presence of NVS.

- Checking app usage (Figure 2c)

The app notifies the patient to update their symptoms every 15 min. If the patient interacts with the app infrequently, the symptom data may be compromised and should be interpreted with caution. Non-compliance with symptom logging is highly unusual [18]. It should be noted that for patients who find it difficult to rate their symptoms, the symptom data should be interpreted with caution and it will be at the discretion of the reporting clinician to gauge the contribution of the patient-reported symptoms to their interpretation of the results.

- Checking artifacts (Figure 2d)

Artifacts are automatically detected and corrected using the onboard accelerometer and validated algorithms in the Gastric Alimetry system [17,23]. Time periods where artifacts were detected are shown by the ‘Artifact Detected’ bar. Excessive artifacts occur when the patient moves, tenses their abdominal muscles, talks and/or laughs, leading to poor data quality or data loss [34,37]. If artifacts are present in >50% of the study period, the test should be interpreted with caution. When artifacts are severe, the data may not be plotted.

- Checking signals (Figure 2e)

The signal traces are consulted when there is uncertainty about whether artifacts have significantly affected the signal. As per Figure 2e, raw signals are shown in the grey line; filtered corrected signals are shown in the blue line. Artifacts appear as deviations where the grey line departs from the blue line, which can range from minor deviations in a small subset of electrodes (e.g., patient touching part of the array) to sharp deviations in all channels (e.g., patient movement). A high rate of artifacts may contribute to a lower registered Gastric Alimetry Rhythm Index (GA-RI), due to small amounts of residual noise that are not corrected.

In our experience, the majority of tests labelled ‘interpret with caution’ can still be interpreted to a satisfactory degree to inform care, such that <1% of Gastric Alimetry tests need to be repeated. The signal and spectral data can be analyzed to determine whether there are sufficient objective data to ascertain a result (e.g., there is a clear visible gastric frequency band and artifacts are limited). It is ultimately a clinical decision to make the final decision to determine the validity and reliability of the results for the diagnostic work-up of the patient. In addition to the steps above, it is also notable that the validated upper body mass index (BMI) limit to the test is currently 35 kg/m^2^ [34]. While gastric activity may be recorded above BMI 35 in many patients, amplitude and rhythm interpretations may become distorted due to signal attenuation through abdominal adipose tissue [24,25,38]. In our database, we have obtained satisfactory test recordings in BMIs up to 60; however, further dedicated studies are in progress to define test performance limitations at higher BMIs.

## 4. Step 2: Spectral Analysis

**Figure 3:**
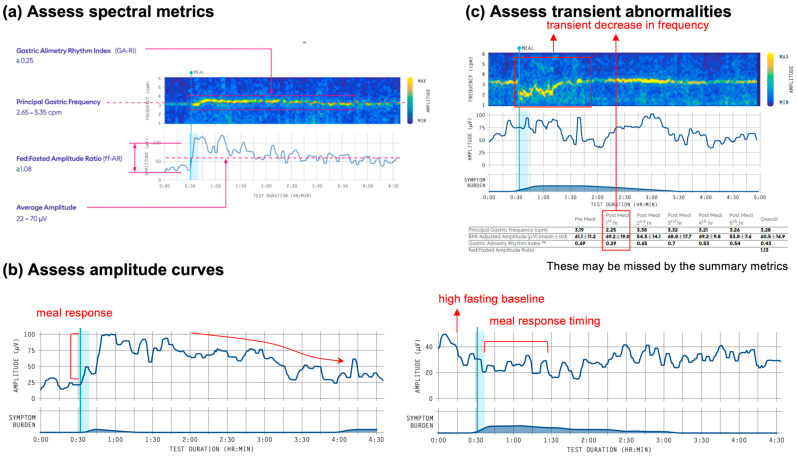
Summary of ‘Spectral Analysis’ guidelines. (**a**) Normal reference intervals for Gastric Alimetry as generated from a large database of healthy adults from diverse demographics (*n* = 110). Four statistically independent spectral metrics are defined with reference to the standardized 4.5 h test protocol: Gastric Alimetry Rhythm Index (GA-RI), Principal Gastric Frequency, Fed:Fasted Amplitude Ratio and Average Amplitude [22]. Reprinted with permission from ref. [25]. Copyright 2023, CC BY 4.0 DEED; (**b**) Assess amplitude curves for meal response: note that the high fasting baseline is a common normal variant; (**c**) Assess for transient abnormalities that may not have been detected in the overall summary metrics.

The spectral analysis produces a spectrogram (graphical representation of the signal amplitude at different frequencies across time) and associated metric tables [17,24]. Four spectral metrics are currently included [24]. These are unique to Gastric Alimetry, designed to specifically correct multiple known pitfalls found to affect the accuracy of legacy EGG test metrics [24]. In addition, reference intervals are available to guide objective evaluation of these metrics, derived from a diverse population of 110 healthy adults (Figure 3a) [22]. Adolescent and paediatric ranges are in development at the time of writing.

- Principal Gastric Frequency (cpm) [Reference interval 2.65–3.35 cpm].

The intrinsic gastric frequency is the dominant feature of the spectrogram. It is observed in normal tests as a distinct horizontal yellow band in the spectrogram and reported in cycles per minute (cpm). Legacy EGG methodologies defined the normal gastric frequency range as 2–4 cpm [36]. The Principal Gastric Frequency is more refined than previous approaches, with normative reference intervals lying within a narrow range of 2.65–3.35 in healthy adults [22]. Small deviations outside this range may be normal, and while females show a slightly higher frequency than males, they are currently assessed using the same range [22].

In legacy EGG, dysrhythmias were defined by frequency abnormalities, with ‘bradygastric’ and ‘tachygastric’ frequencies found in association with diverse gastric disorders [39,40,41]. However, with the robust separation of frequency and rhythm parameters in BSGM [24], together with signal-processing advances [23], isolated deviations in frequency are much less commonly identified in Gastric Alimetry reporting [27,31]. However, frequency elevation (rarely observed to >4 cpm) may be seen in long-term diabetes, hypothesised to reflect autonomic neuropathy [28], and also in vagal injury [26]. Low frequencies (rarely observed to <2.2 cpm) may be associated with intrinsic gastric pacemaker dysfunction or surgical resections [26,42]. Abnormalities may not be sustained throughout the entire meal response and can exist transiently. A Principal Gastric Frequency is not reported when the rhythm stability is low or falls below a critical threshold, indicated by a (-) in the metric table [22,34].

- BMI-Adjusted Amplitude (μV) [Reference interval 22–70 μV]

The amplitude of the gastric signal is corrected for BMI in the Gastric Alimetry system and is reported as microvolts (μV). Based on classical EGG data, it is plausible that sustained high amplitudes (or sustained activity of normal amplitude in the presence of delayed gastric emptying) could be associated with gastric outlet resistance [43,44]; however, further verification of this concept with modern high-resolution approaches is desirable. Low amplitudes may be associated with hypomotility and/or neuromuscular dysfunction [25,27]. It is also important to note that opiates could reduce gastric amplitudes or induce transient dysrhythmias, meaning that these drugs should be ceased at least 24 h prior to Gastric Alimetry testing when possible [45].

- Gastric Alimetry Rhythm Index (GA-RI): [Reference interval ≥ 0.25]

GA-RI is a measure of stability (between 0–1) of gastric activity and quantifies the extent to which activity is concentrated within a normal principal frequency band over time, relative to the residual spectrum [24]. Higher values indicate greater stability, whereas lower values indicate greater spectral scatter. GA-RI is not reported when the amplitude falls below a threshold of <10 μV (indicated by a (-)). A low GA-RI is the biomarker for dysrhythmia and is currently considered to be a key feature indicative of a gastric neuromuscular disorder [27], which likely reflects impaired slow-wave generation and coordination in the presence of underlying ICC network impairment [46,47,48]. Multiple other influences may cause disturbances in gastric rhythmicity, which were recently reviewed in detail elsewhere [13].

- Fed:Fasted Amplitude Ratio (ff-AR): >1.08

A meal response is indicated by the increase in signal power after the test meal compared to before the meal, which is calculated as a ratio of the maximum amplitude in any single 1-h post-prandial period to the amplitude in the pre-prandial period (ff-AR) [24]. During reference range development, it was found that approximately 30% of patients showed a ‘high fasting baseline’ amplitude, such that the reference range cut-off was low (>1.08) (Figure 3b) [22]. The ff-AR metric is therefore not considered a reliable indicator of gastric dysfunction in isolation and is used solely as a supporting metric for an abnormal test in combination with other metrics. 

It should be noted that transient abnormalities in the spectral metrics can also occur (Figure 3c). Such abnormalities will be captured in the hourly reported metrics but may be associated with normal metrics for the overall time period. As there are currently only reference intervals for the overall metrics, assessment of transient abnormalities should be performed on a case-by-case basis. For example, low amplitude or GA-RI before a meal is expected, whereas an hour of high or low frequency activity or low GA-RI immediately after the meal may be indicative of gastric dysfunction, even if it is followed by normal activity.

Based on the initial classification scheme proposed by the BSGM working group [30], five spectral phenotypes have been described: dysrhythmic (GA-RI < 0.25), low-amplitude (BMI-adjusted amplitude < 22 µV), high-amplitude (BMI-adjusted amplitude > 70 µV), high-frequency (frequency > 3.35 cpm); and low-frequency (frequency < 2.65 cpm).

Lastly, it is also of value to assess the amplitude curves, which profile the gastric meal response, per Figure 3b. A typical Gastric Alimetry test shows a post-prandial increase in amplitude that returns toward baseline over the 4 h postprandial period (e.g., Figure 3b; left) [17,22,24]. Meal response curves that show a delayed rise and/or do not return to baseline may be suspicious for gastric dysfunction; however, dedicated studies addressing meal response curves are still awaited before diagnostic utility can be ascertained. In the initial classification scheme proposed by the BSGM working group [31], five spectral phenotypes have been described: dysrhythmic (GA-RI < 0.25), low-amplitude (BMI-adjusted amplitude < 22 µV), high-amplitude (BMI-adjusted amplitude > 70 µV), high-frequency (frequency > 3.35 cpm); and low-frequency (frequency < 2.65 cpm).

## 5. Step 3: Symptoms

The symptom plots are next analysed. When spectral analysis is abnormal, the symptom analysis provides complementary data. When the spectral analysis is normal, specific symptom phenotypes may be identifiable in over half of cases which link to gastric activity patterns [31]. Symptom analysis includes both the pattern and severity of individual symptoms and is optimally conducted according to the following steps.

**Figure 4:**
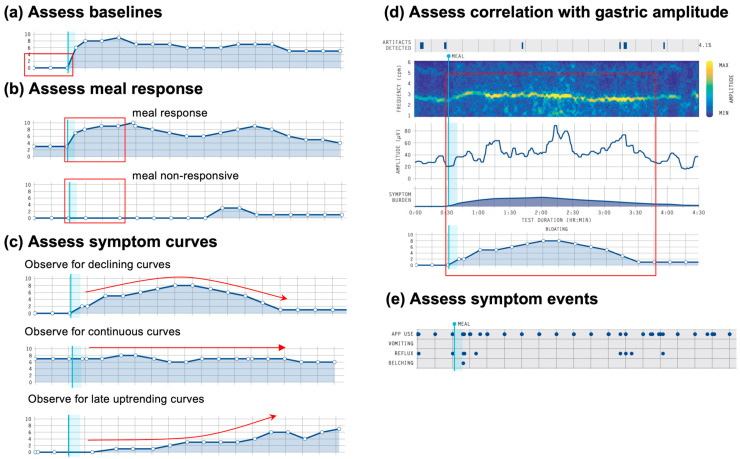
Summary of ‘Symptoms’ guidelines. (**a**) Assess for symptom baseline (red box); (**b**) Assess whether symptoms are meal-responsive or meal non-responsive (red box); (**c**) Assess the symptom curve pattern: declining curve, continuous curve or late uptrending curve (red arrows); (**d**) Assess for correlation between symptom curves and gastric amplitude (red box); (**e**) Assess the timing, type and number of discrete symptom events.

- Assess baselines (Figure 4a), meal response profiles (Figure 4b) and symptom curves (Figure 4c)

It should be noted whether symptoms are present before the meal (including type and severity), followed by an assessment of how the symptoms changed in relation to the meal. The presence of early satiation should be noted as a marker of post-prandial distress [49], which is assessed as a single time-point symptom immediately after the meal (scored out of 10).

Meal-responsive symptoms either increase after the meal and decline over time, or increase with the meal and then remain constant. A symptom curve that increases then decreases in profile (e.g., Figure 4c; top) has been described in association with gastric emptying decay curves, with symptoms abating as food transitions to the small intestine, therefore being a strong indicator that the relevant symptoms have a gastric origin [50]. Alternatively, symptoms may remain relatively continuous throughout the test (Figure 4c; middle), which has been associated with a higher frequency of gut–brain axis (centrally mediated) disorders and vagal neuropathy in published series [27,28]. 

If symptoms trend upwards late into the test, this may suggest a ‘post-gastric’ (small intestine) symptom origin (Figure 4c; bottom), with symptom burden progressively increasing as a greater volume of contents progress beyond the pylorus [25,50]. Symptom curves can also present as mixed profiles, and work is ongoing to further characterise these symptom profiles (refer Tips and Pitfalls). It should be noted whether symptoms are present before the meal (including type and severity), followed by an assessment of how the symptoms changed in relation to the meal. The presence of early satiation should be noted as a marker of post-prandial distress [47], which is assessed as a single time-point symptom immediately after the meal (scored out of 10).

- Assess correlation with gastric activity (Figure 4d)

Next, the symptom and gastric amplitude curves can be assessed together, to determine whether they are correlated, which may indicate visceral hypersensitivity [25]. This assessment can be aided by the total symptom burden bar, which is shown directly under the spectral map in the Gastric Alimetry report (Figure 4d). Symptom curves may also show correlations with transient spectral abnormalities.

- Assess symptom events and correlation with gastric activity (Figure 4e)

Lastly, timing, type and number of symptom ‘events’ (vomiting, reflux and/or belching) should be assessed. The timing of these events can also be correlated with the gastric amplitude.

### 5.1. Emerging Classification Scheme for Symptom Phenotypes

An initial classification scheme for symptom phenotypes has been proposed by a Gastric Alimetry Clinical User Group [30]. Two main categories of symptom profiles are recognized: (a) symptoms related to gastric activity (sensorimotor, post-gastric, and activity-relieved) and (b) symptoms independent of gastric activity (continuous, meal-relieved, meal-induced). Symptom profiles related to gastric activity target gastroduodenal mechanisms such as hypersensitivity, small intestinal pathology, and disorders of gastric accommodation [12,25] For symptom profiles independent of gastric activity, particularly continuous and meal-relieved profiles where there is a high preprandial symptom burden, mechanisms such as brain–gut axis dysregulation or vagal pathologies are more commonly implicated [28,31,51]. Of relevance to cases where symptoms appear high with normal spectral analysis, work is currently underway to integrate psychological-based therapies in routine testing for a disorder of the gut–brain axis [52,53].

Meal-induced and meal-relieved phenotypes are defined by the meal change metric (change in symptoms in relation to the meal stimulus) [54]. A continuous symptom profile reflects a reduced range of symptoms throughout the test (range < 3) and high symptom severity (threshold for the 5th percentile being > 2) [31]. The sensorimotor profile is defined by a symptom–amplitude correlation > 0.5 for a given symptom. The activity-relieved and post-gastric profiles are defined based on the temporal symptom/amplitude curve time-lag (whereby −1 indicates all symptoms occur before all gastric activity, and +1 indicates all symptoms occur after gastric activity). The thresholds for activity-relieved are <−0.25 and >0.25 for post-gastric.

### 5.2. Symptom Correlations with Gastric Activity

**Figure 5:**
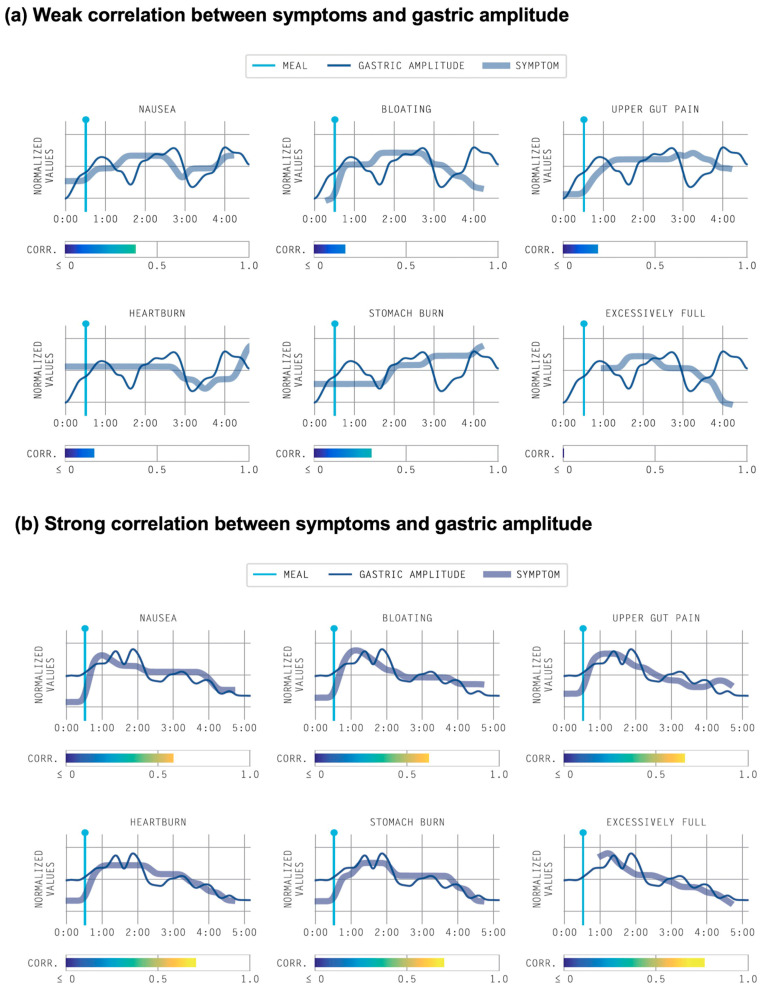
Emerging methods to objectively correlate gastric symptom profiles with gastric activity. (**a**) Example of weak correlation between symptoms and gastric amplitude; (**b**) Example of strong correlation between symptoms and gastric amplitude.

The correlation of symptom curves to gastric amplitude curves is currently performed through a subjective visual assessment and comparison. Therefore, work is currently ongoing to include a standardized objective correlation for all symptoms reported in the Gastric Alimetry test [51]. An example of a suitable approach that is currently being evaluated for inclusion into the Gastric Alimetry report is demonstrated in Figure 5.

## 6. Step 4: Reporting Conclusions

The final step is to summarize the key spectral and symptom features to provide an overall conclusion of test results (‘normal’ or ‘abnormal’). If appropriate, the phenotype/clinical impression should then be suggested per the patient’s clinical context (Table 1) [30,31,51,54]. This classification scheme is currently provisional, with work currently underway by the Gastric Alimetry User Group to formulate the first international consensus.

Phenotyping based on Gastric Alimetry spectral and symptom data is a powerful emerging clinical tool with promising data supporting clinical impact and outcomes [28,33,55]. In addition, these phenotypes may be employed with additional clinical data, including gastric emptying testing [31], to inform management principles. Based on low-resolution EGG data, for example, Koch and colleagues have suggested that pyloric-based interventions may be most suitable for patients with normal spectral profiles but delayed emptying profiles [44,56]. Ongoing work is needed to verify these approaches using high-resolution technologies and further define integrated management pathways.

## 7. Recommended Gastric Alimetry Reporting Format and Considerations (see Box 1 and Box 2)

Based on the above review and discussion, a proforma for Gastric Alimetry reporting is presented in Box 1, with additional considerations presented in Box 2.

Box 1 **Recommended Gastric Alimetry reporting format.**

Test Quality: [Pass/Interpret with Caution]. Impedance good in [all/most/at least half of] channels. [Mild/Moderate/Severe] motion artifacts. [X%] meal completed.

Spectral analysis: The Principal Gastric Frequency is [normal/abnormal] at [X cpm]. A [stable/unstable] GA-RI [>0.25/<0.25] is present. The BMI-adjusted amplitude is normal [=X µV], with a [normal/abnormal] meal response [Fed:Fasted Amplitude Ratio = X]. Consider comments on transient abnormalities and the nature of the gastric amplitude curve.

Symptoms: Symptoms [name symptoms] were [absent/mild/moderate/severe] during the fasting baseline period. Symptoms were [not/weakly/strongly] meal-responsive. Comment on symptom curves and if symptoms were [not/weakly/strongly] correlated with the gastric amplitude curve and/or were continuous throughout the test. Comment on any symptom events and their association with any related spectral observations.

Conclusion: Overall [normal/abnormal] Gastric Alimetry spectral analysis [consider summary of abnormalities]. Symptom profile showed [summary]. Consider [phenotype and clinical impression], as appropriate for the patient’s clinical context.

Box 2 **Additional reporting considerations.**

- The Gastric Alimetry test is currently validated for a BMI of up to 35; interpret results with caution when BMI > 35. In our experience, those with a BMI > 35 will most likely have a BMI-adjusted amplitude within normal ranges since it is challenging to distinguish between low amplitude and signal attenuation due to the abdominal adipose tissue (refer Test Quality).
- Additional comments can be made for any transient spectral abnormalities, e.g., an unstable rhythm index (< 0.25) noted in post-meal 2nd hour.
- If non-standard procedures were used (e.g., alternative meal), comments can be made under ‘Test Quality’, e.g., a non-standard meal was used; interpret with caution.
- It should be noted that Gastric Alimetry does not evaluate all features of gastric function, e.g., gastric accommodation, pyloric function or transit times are not measured using this test.

## 8. Gastric Alimetry Reporting Examples

Examples are provided below of reporting from patient tests, with informed consent granted in all cases for educational use from the Auckland Health Research Ethics Committee.

### 8.1. Example of a Gastric Alimetry Report with a Normal Spectral Analysis and a Sensorimotor Phenotype (Figure 6) [25]

Test Quality: Pass. Impedance ‘Good’ in most channels. Mild motion artifacts (4.1%). 100% meal completed.

Spectral analysis: The Principal Gastric Frequency is normal (overall = 2.77; reference range 2.65–3.35 cpm). A stable rhythm index (overall = 0.63; reference range > 0.25) is present. The BMI-adjusted amplitude is normal (overall = 45.0 µV; reference range: 22–70 µV). The meal response is within normal range (Fed:Fasted Amplitude Ratio = 1.82).

Symptoms: No symptoms noted at baseline. Symptoms are meal-responsive. Mild to moderate nausea, bloating, heartburn and excessive fullness increase after the meal before decreasing at the end of the active gastric period, which appears to be correlated to the gastric amplitude. Ten episodes of reflux are also noted after the meal, which appears to be correlated in timing to the gastric amplitude peak.

Conclusion: Normal Gastric Alimetry spectral analysis with meal-responsive symptoms that correlate with the gastric amplitude. These features fit a sensorimotor phenotype, which may be consistent with a sensitivity and/or accommodation disorder, per associated clinical considerations.

### 8.2. Example of an Abnormal Gastric Alimetry Spectral Analysis (Figure 7) [27,31]

Test Quality: Pass. Impedance ‘Good’ in most channels. Mild motion artifacts (5.7%); 70% of the meal completed.

Spectral analysis: The rhythmic activity is highly unstable post-prandially (overall GA-RI = 0.12; reference range < 0.25). As a result, there is no identifiable overall Principal Gastric Frequency. Where identifiable, the principal gastric frequency is high (3.42 cpm pre meal and 3.46 cpm post-meal 3rd hour). The BMI-adjusted amplitude lies just within the low end of the reference interval (24.6 µV; reference range: 22–70 µV). The meal response is within the normal range (Fed:Fasted Amplitude Ratio = 1.33).

Symptoms: No symptoms are noted at baseline. Mild early satiation (3/10). Symptoms are meal-responsive with moderate excessive fullness and mild bloating reported, which return to baseline by 4 h post-prandially. Two episodes of reflux and moderate belching are also noted.

Conclusion: Abnormal Gastric Alimetry spectral analysis with abnormal gastric rhythm and unrecordable frequencies, accompanied by meal-responsive symptoms. The features may be consistent with a gastric neuromuscular disorder and impaired gastric pacemaking, per clinical correlation.

## 9. Tips, Existing Limitations and Pitfalls

- Artifacts and Colonic Activity

A high sensitivity to artifacts was a major pitfall in the interpretation of classical EGG [57]. This has been addressed in the Gastric Alimetry system through high-resolution electrodes, continuous artifact monitoring, and advanced signal-processing techniques [23,25]. Nevertheless, differentiating artifacts and noise remains an essential consideration in test interpretation.

Both external and intrinsic (biological) noise sources can contaminate the Gastric Alimetry spectral maps, and while these are accounted for automatically in the metric calculations [24], artifacts can still impact both visual and metric interpretations. Large extrinsic artifacts are more obvious in the spectrograms, where they appear as vertical high amplitude bands spanning the whole 1–6 cpm spectrum and are usually reported by the Gastric Alimetry ‘Artifacts Detected’ bar (Figure 8a) [23].

**Figure 7:**
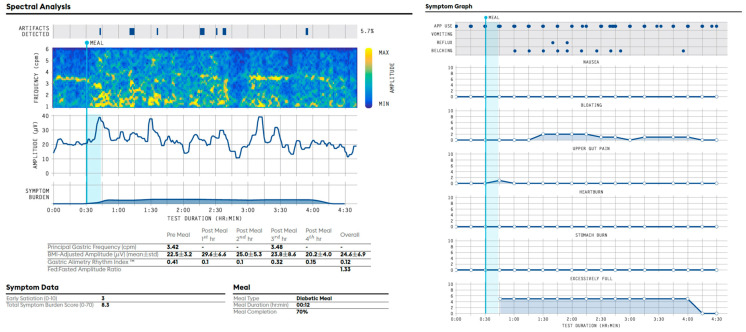
Example of an abnormal Gastric Alimetry spectral analysis.

**Figure 8:**
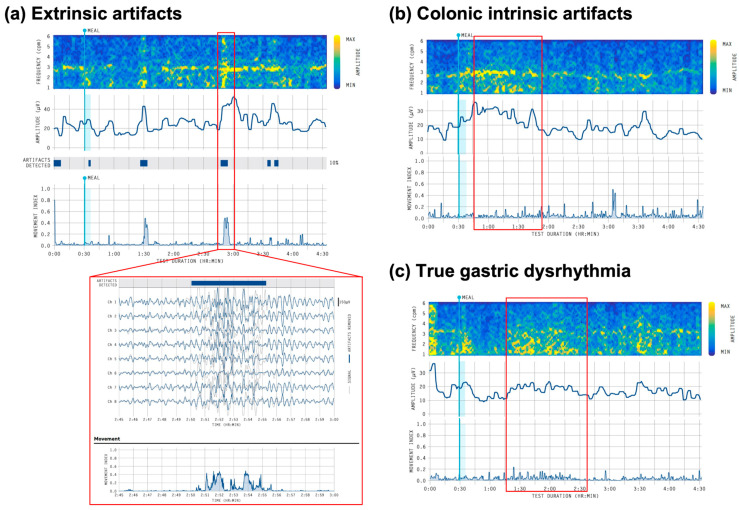
Additional considerations for artifacts. (**a**) Example of an extrinsic movement artifact where they appear as large spikes in amplitude (upper red box) and artifactual signal traces shown in the grey line (bottom red box); (**b**,**c**) Example of colonic intrinsic artifacts showing low-frequency spectral scatter occurring with the Principal Gastric Frequency band and minimal movement artifacts (**b**), and true gastric dysrhythmia showing low-frequency spectral scatter occurring with an absent Principal Gastric Frequency band and minimal movement artifacts (**c**).

Intrinsic noise is more subtle, as it may only affect part of the spectrogram, typically in the low-frequency range (1–3 cpm), and can therefore mimic gastric dysrhythmia [24,58]. As small intestinal activity occurs at a distinctly higher frequency range [59], this low-frequency activity mainly reflects colonic activity, which can occur in a similar range to gastric activity, especially as the transverse colon lies in close anatomical proximity to the stomach [58]. The key criteria for differentiating true gastric dysrhythmia from colonic activity is the concurrent presence or absence of a Principal Gastric Frequency (Figure 8b,c) [24]. If a Principal Gastric Frequency is concurrently present, then low-frequency spectral scatter is suspected to be colonic activity; if it is absent or patchy, then it can be assumed that the gastric activity is discoordinated and a true dysrhythmia is present [24]. A fragmented or intermittent Principal Gastric Frequency band with interspersed spectral scatter is particularly indicative of a gastric abnormality (e.g., Figure 7).

- Application of Normative Reference Intervals

The reference intervals for spectral analysis discussed above were generated for participants aged ≥18 years with BMI < 35 kg/m^2^, where >50% of the meal is consumed during the test and < 50% of the test duration is affected by artifacts [22]. Several considerations should be remembered in their application, as with all medical reference intervals [60]. These intervals serve as a guide for patient phenotyping and are not ‘diagnostic’ categories in themselves. Distributions between patients and controls may overlap, and ultimately it should be remembered that the Gastric Alimetry test is a diagnostic aid that requires integration with clinical knowledge of the individual patient by the reporting clinician [25]. A high-calorie meal (e.g., 482 kcal; 5 g of fat, 45 g of carbohydrate, 10 g of protein, 7 g of fiber) was chosen to provide sufficient stimulus so that symptoms can be evoked in diverse gastroduodenal populations [22,27]. In a database of >2000 cases, we have found that around 5% of participants were unable to complete at least 50% of the test meal. Normative reference intervals for smaller meals are currently being investigated and may be useful for these participants with lower meal tolerances. Future studies assessing the use of Gastric Alimetry with nutrient drink tests could also be valuable to measure gastric hypersensitivity and/or impaired gastric accommodation, including in functional dyspepsia [61,62].

- Validation of symptom profiles

Acceptable correlations have been shown between the app-based symptoms and PAGI-SYM/PAGI-QOL questionnaires [18], allowing for differences in time-of-test symptom logging vs. PAGI questionnaires that test symptom recall over a two-week period. Ongoing work is providing further validation for a mechanism-based approach to objectively classify app-based symptom profiles [51,54].

- Mixed Profiles

Another challenge affecting Gastric Alimetry test interpretation is mixed or non-specific test profiles. Currently, >60% of tests yield a specific diagnostic phenotype [31], with future advances expected to bring increased objectivity to symptom phenotyping while raising this yield to 80%+ [51]. It should also be noted that pathophysiologies contributing to chronic gastroduodenal symptoms are diverse [7,8,9] and may overlap. In the absence of a specific phenotype or mixed profile arising from the test, evaluation of a patient’s dominant symptoms is helpful to inform therapeutic directions, together with reference to other complementary gastric function tests such as gastric emptying [31].

## 10. Discussion

This paper has reviewed the current literature underlying Gastric Alimetry and BSGM, in order to offer a systematic interpretation guide for clinical test usage. The recommended reporting format consists of four sections: Test Quality, Spectral Analysis, Symptoms and Conclusions. A synoptic reporting format and template have been presented. Technical and clinical considerations have been reviewed for each section, in order to provide readers with the necessary evidence to interpret tests with confidence. The resultant methodology is already being applied in clinical practice and research by the authors and is now recommended for other users adopting the test.

With over 2000 Gastric Alimetry tests accrued, test interpretation is likely to continue to evolve rapidly as new phenotypes and clinical evidence emerge. An expanded range of symptom phenotypes is currently consolidating, with more objective criteria, resulting in three major categories: (i) spectral abnormalities; (ii) symptom profiles linked to gastric activity; and (iii) symptom profiles independent of gastric activity [30,51,54]. Evidence from increasingly large cohorts has shown potential for this approach to distinguish neuromuscular, sensorimotor/hypersensitivity, and gut–brain abnormalities among other pathophysiologies; with anxiety and depression most strongly linked to phenotypes that are independent of gastric activity [27,31,51].

Example studies include an evaluation of 32 type 1 diabetics and 32 matched controls, with distinct phenotypes showing correlations with symptoms along with glycemic control, peripheral neuropathy and psychological co-morbidities. A study of 43 NVS patients and 43 matched controls distinguished between two distinct NVS subgroups: 31% with abnormal BSGM correlating with symptoms and 62% with normal BSGM correlating with increased psychological comorbidities [27]. In a different study of 75 patients with chronic gastroduodenal symptoms, Gastric Alimetry demonstrated a 2.7 times higher diagnostic yield compared to gastric emptying scintigraphy [31]. Moreover, the evaluation of 210 patients with chronic gastroduodenal symptoms resulted in over 80% being classified into distinct mechanism-based phenotypes, which correlated with patient-reported symptoms, quality of life and psychological factors [51]. New visualizations and metrics could aid in the understanding and objective evaluation of these phenotypes, e.g., Figure 5.

Other promising directions include incorporating a gut–brain health questionnaire into the Gastric Alimetry App and Report, for patients to complete during the test. This idea has been strongly supported in surveys of both clinical and patient users [52], reflecting the growing awareness of gut–brain axis linkages as a determinant in chronic gastrointestinal symptoms [49,63]. In addition, work continues to evaluate and validate spatial patterns of gastric activity [17,19,64], which have been linked to symptom profiles in CNVS, gastroparesis and functional dyspepsia in research studies in both adults and children [19,20,65]. Furthermore, gastrointestinal neuropeptides and hormones have been implicated in modulating gastric function, which may lead to the onset of symptoms [66]. Investigating the role of these small molecules in controlling gastric activity serves as another area of future research.

The time taken to interpret a Gastric Alimetry test using the reviewed system can vary according to the complexity of the case. However, average timings have been evaluated. Upon completion of the Gastric Alimetry test, the data are transferred to the HIPPA-compliant Alimetry cloud. The clinician retrieves the report from the cloud and interprets it over an average duration of approximately 35 min. Reviewing the results with the patient takes an average of 15 min, and additional patient management documents take a further 10 min.

In conclusion, recent advances in BSGM, digital symptom profiling, and big-data analytics have presented a strong foundation for the entry of Gastric Alimetry into the diagnostic toolkit for chronic gastroduodenal symptoms. It is anticipated that the interpretation methodology reviewed here will support the standardized and evidence-based adoption of Gastric Alimetry into practice.

## Figures and Tables

**Figure 6:**
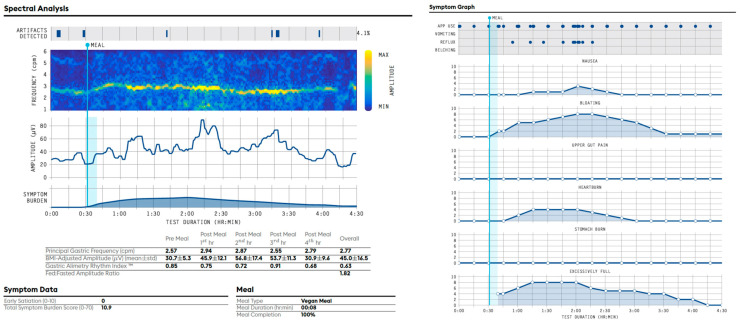
Example of a normal Gastric Alimetry spectral analysis, with a ‘sensorimotor’ phenotype profile.

**Table 1:** Phenotypes to consider based on the spectral and symptom features per patient’s clinical context from the classification scheme [30]. Note that features may overlap.

Feature	Criteria	Pathophysiology to Consider *
Dysrhythmic	GA-RI < 0.25	Gastric neuromuscular disorder Dysrhythmic states
Low-amplitude	BMI-adjusted amplitude < 22 µV	Hypomotility Myopathy Gastric neuromuscular disorder or myopathy
High-amplitude	BMI-adjusted amplitude > 70 µV	Gastric outlet resistance
High-frequency	Frequency > 3.35 cpm	Long-term diabetes Vagal neuropathy or injury
Low-frequency	Frequency < 2.65 cpm	Impaired pacemaker function Resection of primary gastric pacemaker
Sensorimotor profile	Normal spectral analysis Meal-responsive symptoms that correlate with gastric amplitude	Hypersensitivity and/or impaired accommodation disorder
Continuous profile	Normal spectral analysis Non-meal-responsive symptoms that persist at a high severity throughout test, including before meal	Disorder of gut–brain axis or vagal neuropathy or non-gastric cause
Post-gastric profile	Normal spectral analysis Symptoms trend upwards late in the test as gastric amplitude decays	Consider small bowel pathophysiology

* Note: phenotypes are currently emerging and therefore considered provisional at the time of writing. For further discussion of pathophysiological associations, refer to [25,27,28,31].

## Data Availability

The data presented in this study are available on request from the corresponding author. The data are not publicly available due to ethics.
